# Organ-specific accumulation of selenium and mercury in Indo-Pacific bottlenose dolphins (*Tursiops aduncus*)

**DOI:** 10.1186/s13028-021-00607-w

**Published:** 2022-01-27

**Authors:** Masumi Marumoto, Mineshi Sakamoto, Masaaki Nakamura, Kohji Marumoto, Shozo Tsuruta

**Affiliations:** 1 National Institute for Minamata Disease, 4058-18, Hama, Minamata, Kumamoto 867-0008 Japan; 2 Department of Dental Material Science, School of Dentistry, Aichi Gakuin University, 1-100 Kusumoto-cho, Chikusa-ku, Nagoya, Aichi 464-8650 Japan

**Keywords:** Distribution, Electron probe microanalysis, Inorganic mercury, Methylmercury, Trace elements

## Abstract

Delphinids are top ocean predators and accumulate high concentrations of mercury (Hg) through the food chain, particularly in organs such as liver and kidney, although the proportion of methylmercury (MeHg) is relatively low due to the demethylation process. Total mercury (T-Hg) levels in marine mammals have been shown to correlate with selenium (Se) concentrations, and ingested MeHg that is demethylated may be present in tissues as mercury selenide (HgSe). In this study, we determined T-Hg, MeHg and Se concentrations of three Indo-Pacific bottlenose dolphins (*Tursiops aduncus*), and we used the individual with the highest Hg concentration for electron probe microanalysis to assess the co-localization of Hg and Se in the tissues. By electron probe microanalysis, we found that Hg and Se were co-localized in large granules in hepatic Kupffer cells and in small granules in hepatocytes. The analysis suggested that MeHg was demethylated in hepatocytes and then phagocytosed by Kupffer cells. In the kidney, Hg and Se were co-localized in the glomerular capillary wall and in interstitial blood vessel walls. Hg and Se were also co-localized in the cytoplasm of large neurons and in glial cells in the cerebrum. Divalent Hg and HgSe cannot cross the blood–brain barrier, suggesting that MeHg is demethylated in the dolphin brain and that binding to Se suppresses Hg toxicity.

## Findings

Delphinids are top ocean predators and accumulate mercury (Hg) through the food chain [1, 2]. Hg accumulates in high concentrations, particularly in liver and kidney [3], although the proportion of methylmercury (MeHg) is relatively small (10% or less) due to a demethylation process [4]. Total mercury (T-Hg) levels are typically co-localized and correlated with selenium (Se) concentrations [4] because Se has a high affinity for Hg [5, 6]. Se has been shown to protect against MeHg toxicity in whales and experimental animals [7, 8]; in some species, ingested MeHg is converted to inorganic mercury (I-Hg), an insoluble Hg selenide mineral ‘termed’ tiemannite [7, 9]. Therefore, in the liver, Hg and Se are present at a molar ratio of 1:1 in various whale species [10, 11].

Photoemulsion histochemistry and autometallography method can be used to detect I-Hg in clinical samples [12, 13]. However, as there is no way to visualize Se bound to I-Hg, histological localization of Se has not been elucidated. Electron probe microanalysis visualizes the distribution of trace elements in tissue sections [14, 15], and has been used to detect the distribution of trace elements in the tissues of patients with primary biliary cirrhosis, Minamata disease, Wilson’s disease, and occupational lung diseases [14–17]. Electron probe microanalysis can localize trace elements at the level of specific cell types [14–17]. Sakamoto et al. reported the localization of Se and Hg in the skeletal muscle of a dolphin [8], but their co-localization in organs other than skeletal muscle has not been clarified. In this study, we analyzed the Hg and Se concentrations in the tissues of three Indo-Pacific bottlenose dolphins (*Tursiops aduncus*) and we used the individual with the highest Hg concentration for electron probe microanalysis to assess the co-localization of Hg and Se in the tissues.

**Fig. 1:**
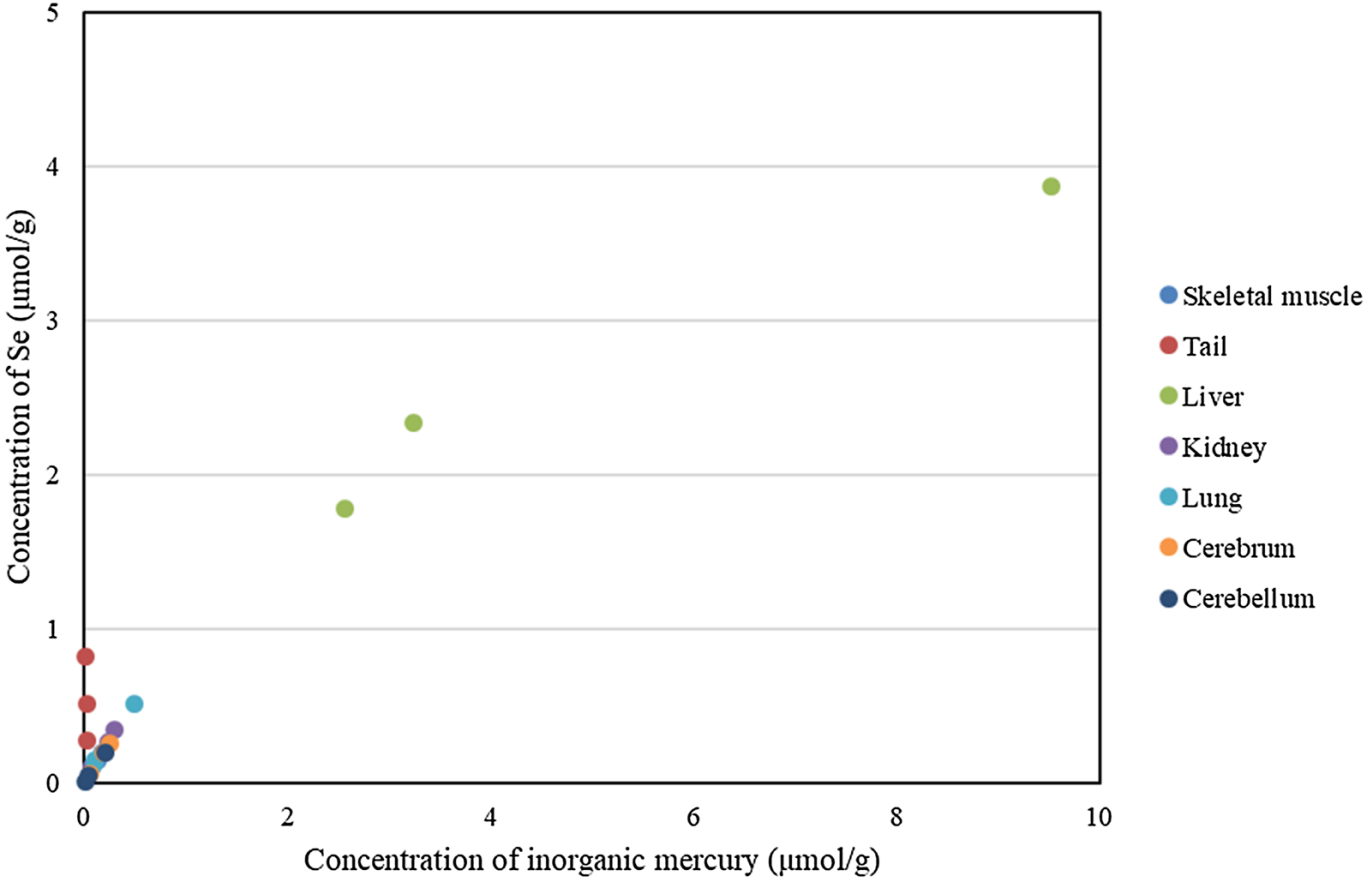
The relationship between I-Hg and Se contained in organs is shown. Hg levels in liver are shown to be higher than in other organs

Cryopreserved tissue samples of liver, kidney, lung, skeletal muscle, tail (bone-free skin and connective tissue), cerebrum, and cerebellum of 3 adult female dolphins captured as food in the Pacific Ocean along the coast of main island of Japan in 2011 were used for analyses. T-Hg concentrations were determined by cold vapor atomic absorption spectrophotometry, and MeHg concentrations were measured using gas-chromatography-electron capture detector, according to previously described methods [18]. The I-Hg concentration was calculated subtracting methylmecury from the T-Hg concentration. Se concentrations were measured by an inductively coupled plasma mass spectrometer equipped with a collision cell (Agilent 7500ce; Agilent Technologies, Santa Clara, CA, USA) by IDEA Consultants, Inc. (Shizuoka, Japan). T-Hg in a standard reference material, DORM-2 (dogfish muscle; National Research Council of Canada, Ottawa, ON, Canada), was measured as a quality control, and the measurements were within the certified range of 4.64 ± 0.26 μg/g. The same standard reference material was used to measure MeHg, which was within the certified range of 4.47 ± 0.032 μg/g. NIST 1577 (bovine liver; Gaithersburg, MD, USA) was used as a quality control for Se measurement, and the measurements were within the certified range of 0.73 ± 0.06 μg/g. T-Hg, MeHg and I-Hg concentrations and the relationship between molar inorganic Hg and Se in organs of dolphins are shown in Table 1 and Fig. 1, respectively. The molar ratios of inorganic Hg to Se is shown in Table 2. Hg concentrations were highest in the liver, and the molar ratios of Hg to Se were close to 1 in most of the organs. In the tail the molar ratios were between 0.02 and 0.08, and only in the liver the molar rations where higher than 1.

**Table 1:** Concentrations of total mercury (T-Hg), methylmercury (MeHg) and inorganic mercury (I-Hg) in tissues from bottlenose dolphins

	Case 1			Case 2			Case 3		
	T-Hg	MeHg	I-Hg	T-Hg	MeHg	I-Hg	T-Hg	MeHg	I-Hg
Liver	1930	13.0	1917	662	11	651	534	17	517
Kidney	66.2	4.8	61.4	51.3	3.4	47.9	21.6	5.7	15.9
Lung	104	3.0	101	22.3	2.3	20	25.6	3.4	22.2
Cerebrum	53.3	2.6	50.7	41.1	2.3	38.8	14.8	3.2	11.6
Cerebellum	43.2	2.1	41.1	3.6	1.6	2.0	13.5	3.5	10
Skeletal muscle	50.6	13.8	36.8	33.8	7.9	25.9	25.6	10	15.6
Tail	12.8	6.6	6.2	7.9	3.1	4.8	15.7	11.3	4.4

Units are microgram per gram of wet weight

**Table 2:** Molar ratio of I-Hg to Se in tissues from bottlenose dolphins

	Case 1	Case 2	Case 3
Liver	2.46	1.39	1.45
Kidney	0.89	0.90	0.72
Lung	0.98	0.78	0.74
Cerebrum	1.00	1.00	1.07
Cerebellum	1.04	0.93	0.96
Skeletal muscle	0.93	0.9	0.83
Tail	0.06	0.08	0.02

For histopathological examination, cryopreserved tissues of the dolphin with the highest Hg concentration (case 1) were fixed in 10% neutral buffered formalin. After fixation, a paraffin block was prepared according to a conventional method with ethanol and xylene and sliced into 3-μm serial sections with a microtome and stained with hematoxylin and eosin. There were no pathological findings in any of the tissues examined. Serial sections were affixed to a carbon sample stage (Niigata Science, Niigata, Japan), deparaffinized, and dried. The specimens were sputter-coated with carbon prior to elemental analysis. Scanning electron microscopy was performed to assess morphological changes, and energy-dispersive X-ray spectroscopy was conducted to determine the elemental composition using an electron probe microanalyzer (JXA-8530F, JEOL, Tokyo, Japan) with an acceleration voltage of 15 kV. Next, we used electron probe microanalysis for elemental mapping of Hg, Se, zinc (Zn), sulfur (S), iron (Fe), and copper (Cu) with 256 × 256 pixel mapping. In order to clarify the pathological image, mapping of amino nitrogen and nucleotide phosphorus was carried out. The acceleration voltage and beam current were set to 15 kV and 0.38 μA, respectively.

**Fig. 2:**
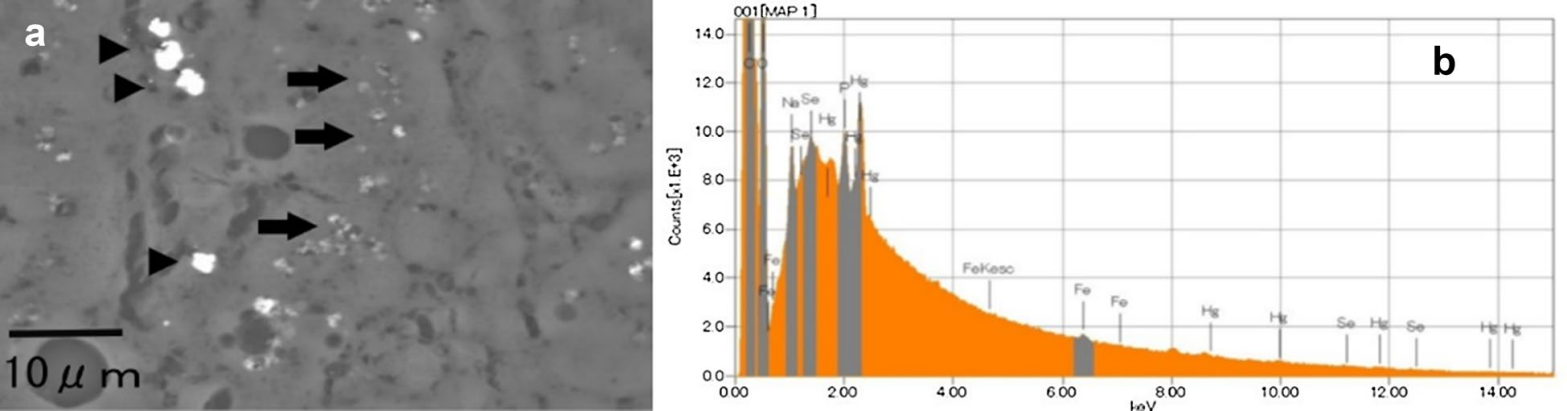
**a** Compositional image in backscattered electron mode of white granular materials, indicating element aggregation in hepatocytes (arrows) and Kupffer cells (arrowheads) of the liver. **b** Energy-dispersive X-ray spectroscopy showed that these white granules contained Hg and Se

**Fig. 3:**
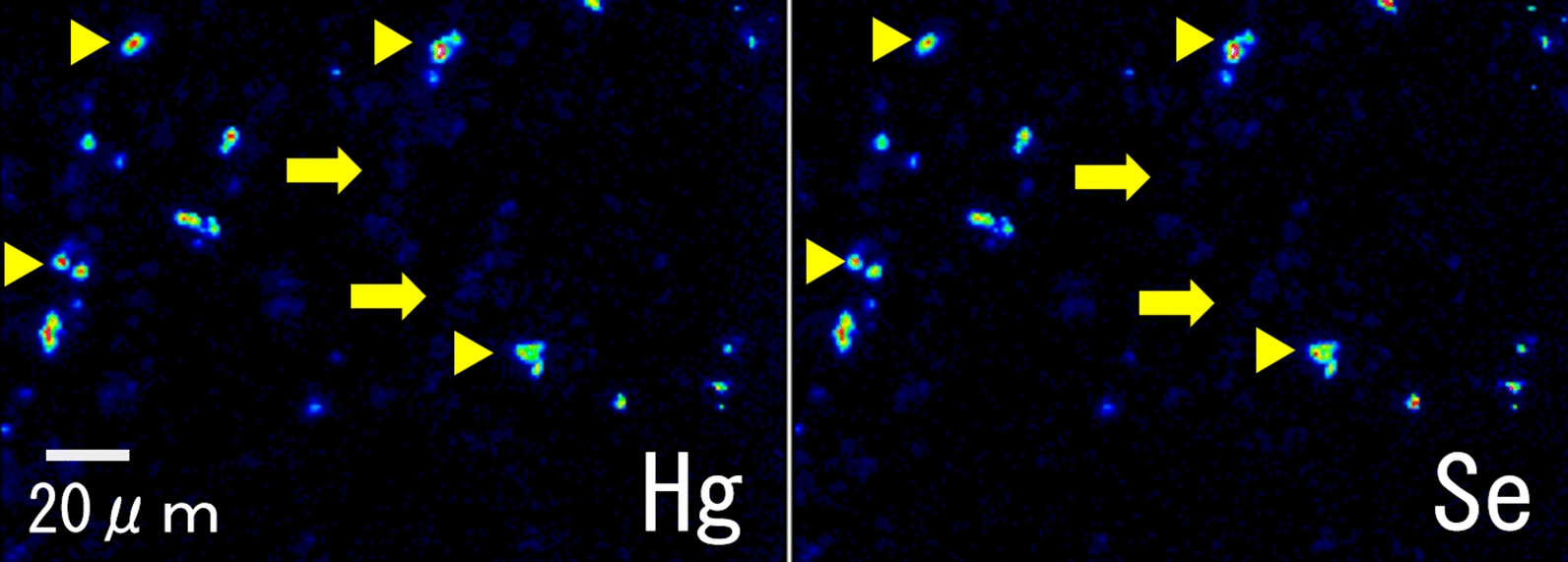
Elemental mapping of mercury (Hg) and selenium (Se) deposited in hepatocytes (arrows) and Kupffer cells (arrowheads) of the liver. The distribution of these two elements was almost identical. Higher concentrations of Hg and Se were observed in Kupffer cells than in hepatocytes

**Fig. 4:**
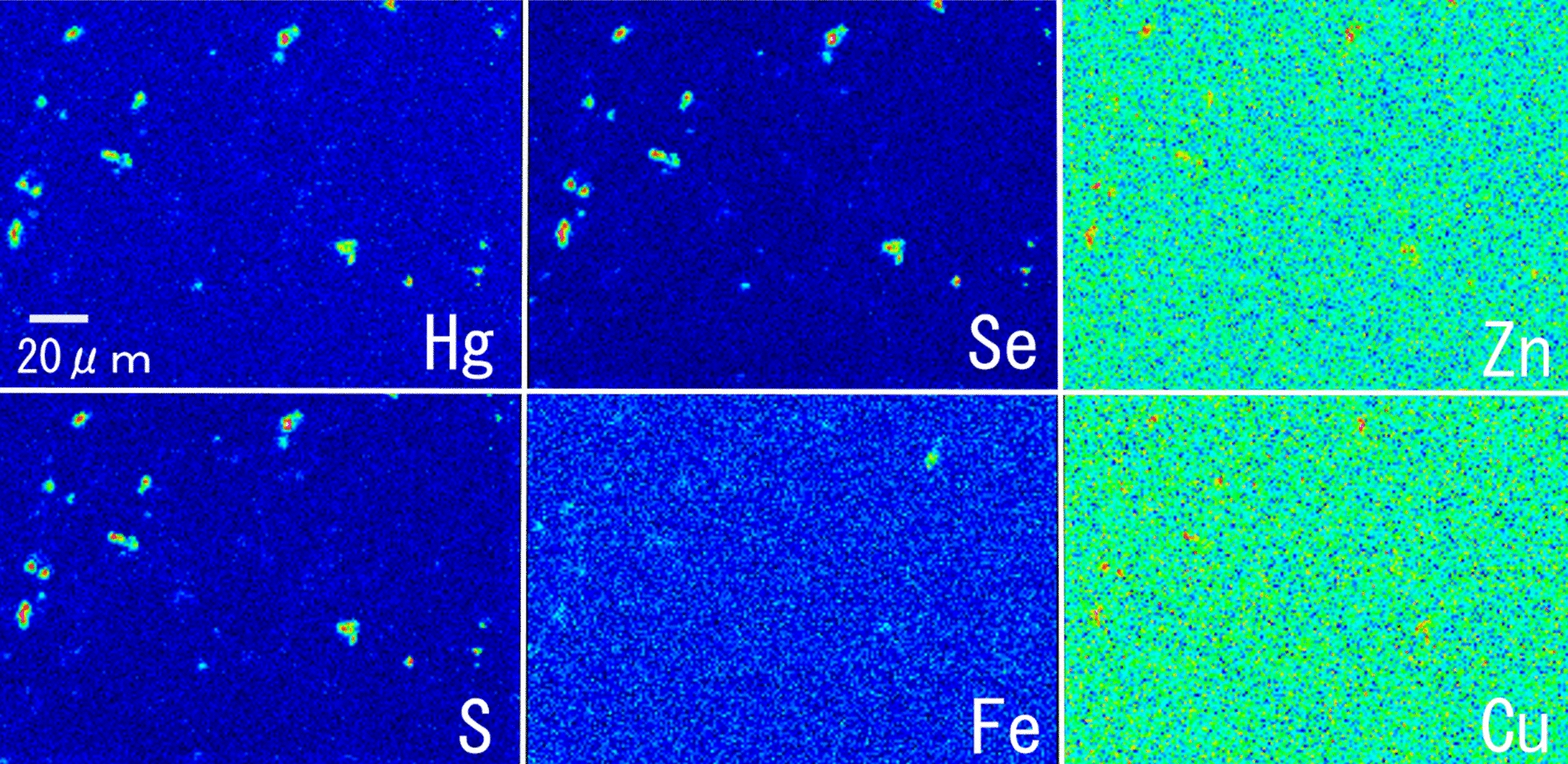
Elemental mapping identified deposits of mercury (Hg), selenium (Se), zinc (Zn), sulfur (S), iron (Fe), and copper (Cu) in Kupffer cells of the liver

**Fig. 5:**
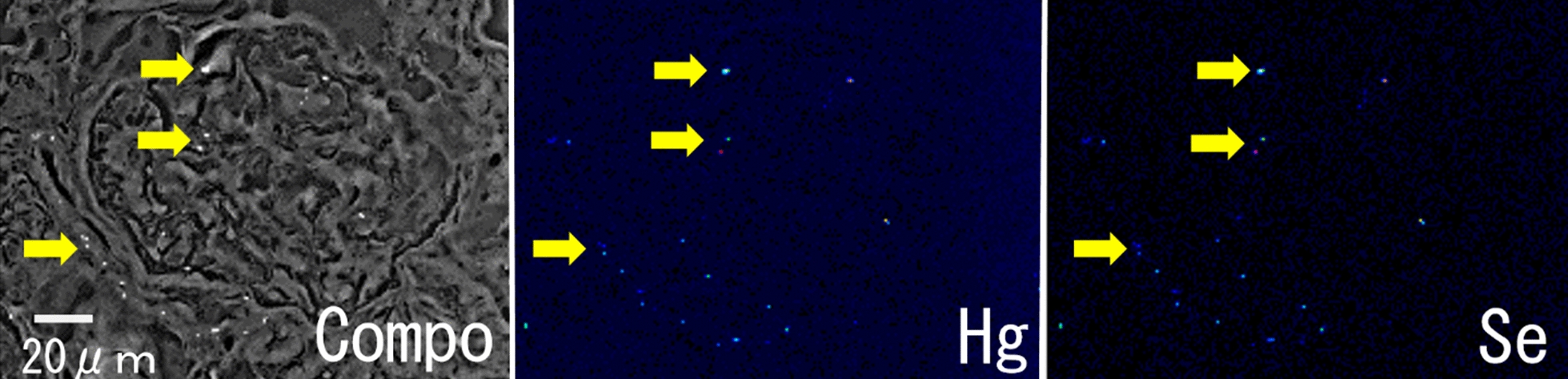
Compositional image in backscattered electron mode (Compo) of white granular materials in the kidney, indicating element aggregation in the glomerular walls and the interstitium (arrows). Elemental mapping shows the aggregation of mercury (Hg) and selenium (Se), which were co-localized

**Fig. 6:**
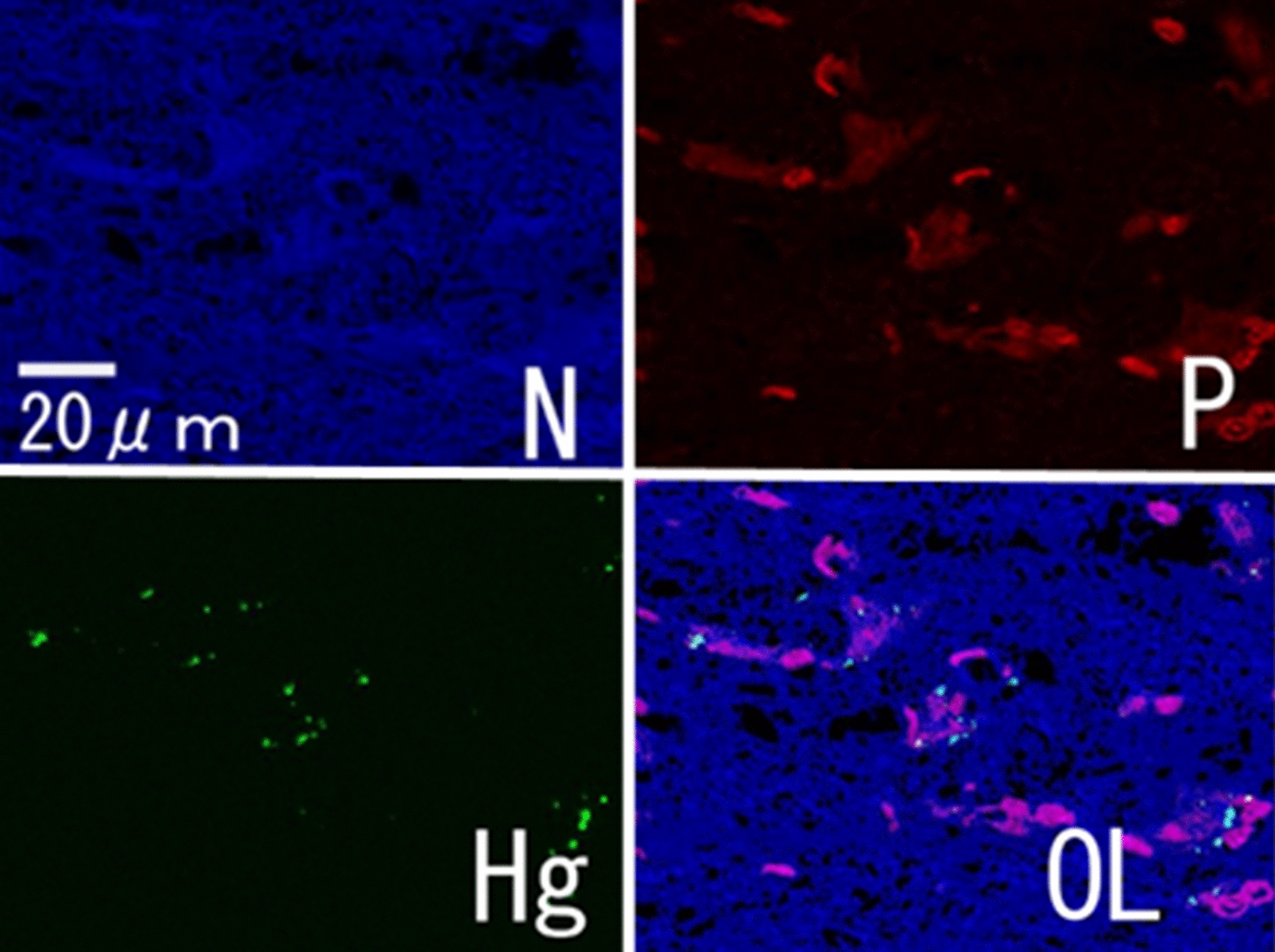
Electron probe microanalysis mapping of nitrogen (N), phosphorus (P), mercury (Hg), and an overlapping (OL) image in the cerebrum. Hg deposition in the cerebrum was observed in the cytoplasm of large neurons (light blue granules in the OL image)

**Fig. 7:**
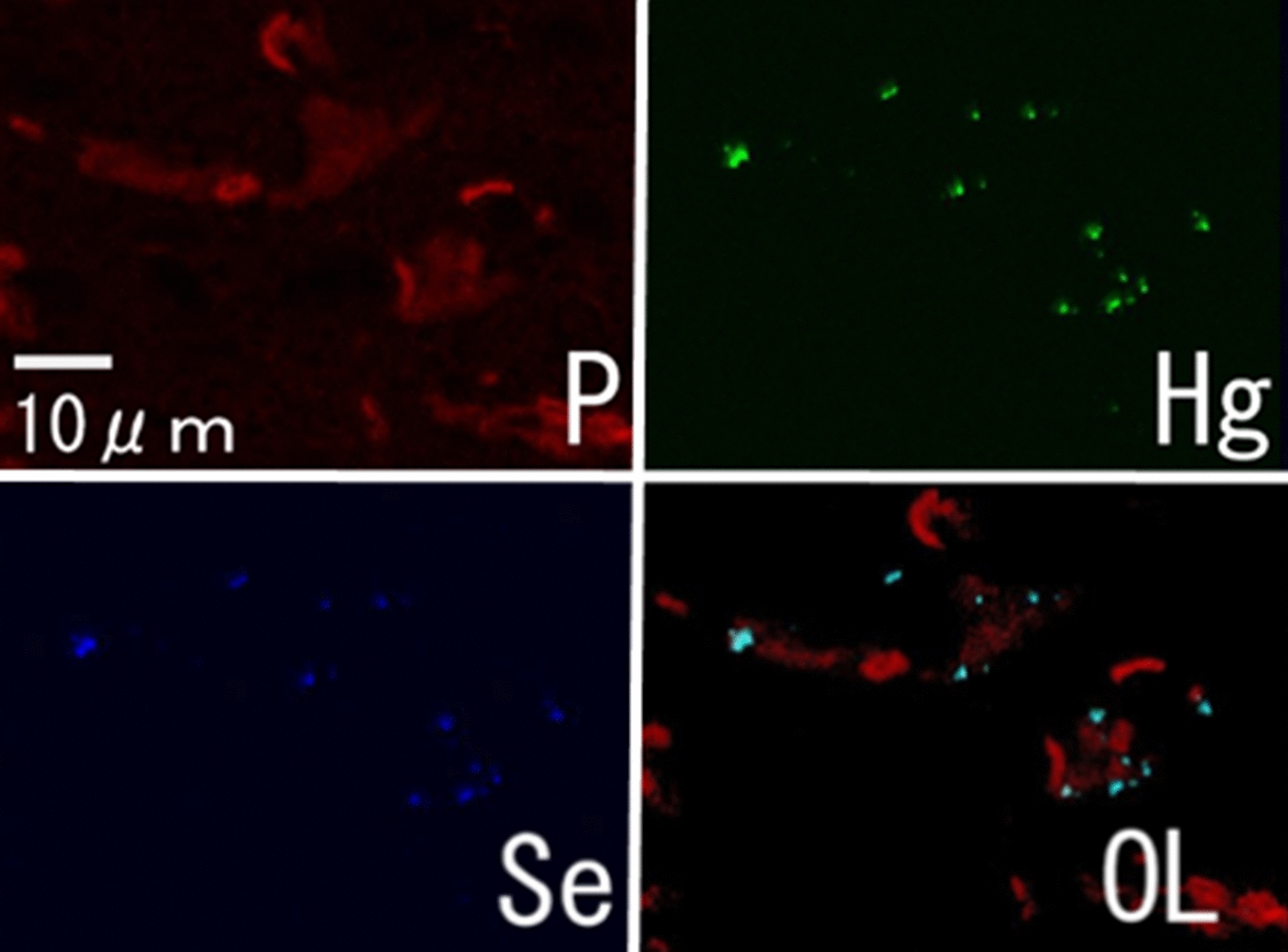
Electron probe microanalysis mapping of phosphorus (P), mercury (Hg), selenium (Se), and an overlapping (OL) image in the cerebrum. Hg and Se were co-localized in the cerebrum (light blue granules in the OL image)

Figure 2a shows a compositional image of the liver in backscattered electron mode. The white granules suggesting metal accumulation were observed in large numbers as large granules in Kupffer cells and as fine granules in hepatocytes. Energy-dispersive X-ray spectroscopy showed Hg and Se in hepatocytes (Fig. 2b) and Hg, Se, Zn, S, Fe, and Cu in Kupffer cells (data not shown). Elemental mapping also showed that Hg and Se were deposited in hepatocytes, and their distribution was almost identical (Fig. 3). Large aggregation of Hg and Se were observed in Kupffer cells than in hepatocytes. Hg, Se, Zn, S, Fe, and Cu were deposited in Kupffer cells (Fig. 4). Figure 5 shows a compositional image of the kidney in backscattered electron mode, showing deposition of white fine granules on the glomerular capillary wall and the interstitium. Energy-dispersive spectroscopy showed the granules to be composed of Hg and Se (data not shown). Fine white granules suggesting metal deposition were observed in the interstitium but not in the renal tubules (Fig. 5). Elemental mapping at the same site identified the elements deposited in the glomeruli as Se and Hg (Fig. 5). Metals were also observed in the large and small blood vessel walls of the kidney. In the lung, fine granules were identified as Hg and Se, suggesting element accumulation in the interstitial but not in alveolar macrophages. In the cerebrum, Hg and Se were observed in glial cells and the cytoplasm of large neurons. Figure 6 shows electron probe microanalysis mapping and overlap of nitrogen, phosphorus, and Hg; Hg deposition in the cytoplasm of cerebral large neurons was observed. Figure 7 shows electron probe microanalysis mapping and overlap of phosphorus, Hg, and Se; the images indicate the relative distribution of Hg and Se, which were co-localized (light blue granules), suggesting that they were present as mercury selenide (HgSe). In the cerebellum, Hg and Se were observed in glial cells. In the tail, no Hg or Se was observed. In skeletal muscle, Hg and Se were deposited around the nucleus.

When MeHg is demethylated in hepatocytes and it is converted to I-Hg, it cannot pass easily through the brain-blood barrier [19]. However, we found Hg and Se in neurons and glial cells, and presume that these cells perform demethylation in the dolphin brain. Considering the observed absence of MeHg poisoning in dolphins [19], our results suggest that this feature may be attributable to a high demethylation capacity in the brain. Aggregation of HgSe was previously reported in Kupffer cells from dolphins [7]. Kupffer cells are derived from bone marrow monocytes and contain numerous lysosomes and phagocytic matter; they act as macrophages to remove bacteria and phagocytose substances metabolized in hepatocytes. In this study, we found S co-localized in Kupffer cells with Hg and Se. S is present in various cells in vivo, and has high affinity to metals. Kupffer cells typically contain other metals that can bind to S, but Se has a higher affinity to Hg than to S [20], suggesting that excess Hg that could not be bound to Se may be bound to sulphur in Kupffer cells. We did not find Hg and Se in alveolar macrophages and other phagocytic cells, with the exception of Kupffer cells. This suggests that only hepatocytes retain the demethylation function, and Kupffer cells only phagocytize local and demethylated Hg and Se. In the lung and kidney, Hg and Se were observed in the interstitium and blood vessel walls. In contrast, in humans exposed to MeHg, Hg and Se are deposited in the proximal and distal tubules of the kidney [12]. Therefore, the observed differences between dolphins and humans may indicate differences in Hg and Se deposition among distinct mammalian orders, although we tested organs from only one dolphin. In skeletal muscle cells, Hg and Se were deposited around the nucleus. Because of the abundance of mitochondria around the nucleus, mitochondrial demethylation may occur, and electron microscopy is required to test this hypothesis.

We showed that Se and Hg are co-localized not only in liver and kidney but also in other organs such as brain, skeletal muscle and lung. This suggests cellular demethylation of MeHg and binding of I-Hg to Se to form HgSe throughout the body, promoting further accumulation of Hg and Se.

## Data Availability

The datasets used and analyzed during the current study are available from the corresponding author upon reasonable request.
